# Level of Fruit and Vegetable Intake and Its Relationship with Risk for Malnutrition in China’s Adult Labor Force: China Nutrition and Health Surveillance, 2015–2017

**DOI:** 10.3390/nu15061431

**Published:** 2023-03-16

**Authors:** Qiya Guo, Hongyun Fang, Liyun Zhao, Lahong Ju, Xiaoli Xu, Dongmei Yu

**Affiliations:** 1 National Institute for Nutrition and Health, Chinese Center for Disease Control and Prevention, Beijing 100050, China; 2 NHC Key Laboratory of Trace Element Nutrition, National Institute for Nutrition and Health, Chinese Center for Disease Control and Prevention, Beijing 100050, China

**Keywords:** fruits, vegetable, labor force, BMI, China

## Abstract

The purpose of this study was to analyze the fruit and vegetable intake status and examine both potential risk and protective action factors in the Chinese labor force population, while investigating the association between fruit and vegetable intake and malnutrition in this population. Data were derived from a population-based cross-sectional survey, the China Nutrition and Health Surveillance, 2015–2017. Sociodemographic information, physical measurements, and dietary intake data were collected. A total of 45,459 survey respondents aged 18–64 years old were included in the analysis. Fruit and vegetable consumption data were assessed by a food frequency questionnaire (FFQ), and the average daily intake was calculated. In 2015, the daily intake of fresh fruits, fresh vegetables, and combined fruits and vegetables among the Chinese labor force was 64.3 g, 210.0 g, and 330.0 g (median), respectively. Compared with the Dietary Guidelines for Chinese Residents (2022), 79.9% and 53.0% were at risk of inadequate fruit and vegetable intake, while 55.2% were at risk of inadequate intake of combined fruits and vegetables compared with the WHO recommendation. Multivariate logistic regression analysis showed that female gender, higher educational level, and higher income were protective factors for adequate fruit intake, while increasing age and living in the southern region were protective factors for adequate vegetable intake. The results confirmed that increasing vegetable intake helped urban labor force maintain normal BMI and control overweight. Increased fruit consumption may reduce the risk of underweight, but no clear negative association with overweight and obesity was observed. In conclusion, the consumption of fresh fruits and vegetables by the Chinese labor force was inadequate, especially for fruits. Interventions are needed to promote the daily intake of fruits and vegetables in this population. In addition, further in-depth studies in this area are recommended in populations with different health status.

## 1. Introduction

Labor resource refers to the total population of a country within the range of working age and capable of working in a certain period, and health status is one of the most important determinants of labor quality. Research shows that there is a significant positive correlation between the health status of the labor force and economic growth, especially in developing countries [1,2]. Improving the quality of the workforce can also prevent and reduce the future prevalence of chronic diseases among the elderly population and mitigate the declining labor supply due to increasing aging. According to the data of the Sixth Census in 2010, China’s labor force accounts for 70.14% of the total population and is the most important foundation for China’s social development [3]. In recent years, with the rapid development of social economy and medical and health technology, the nutrition and health status of China’s labor force have been greatly improved. However, at the same time, nutrition-related chronic diseases such as overweight, obesity, diabetes, dyslipidemia, hypertension, and cardiovascular diseases are on the rise due to inappropriate dietary structure [4,5]. Therefore, as a developing country, China is now inevitably facing the double burden of malnutrition (undernutrition and overnutrition), which is closely related to dietary structure.

As an important part of healthy diet, increasing the daily intake of fresh fruits and vegetables is an important link to improve the health status of China’s labor force. Fresh fruits and vegetables are the primary dietary source of a wide range of chemicals and nutrients, and have been identified as one of the key dietary factors in the global disease burden [6]. Previous studies have shown that inadequate intake of fruit and vegetable may result in illness, disability, and death. In 2017, 11.4% of all disease deaths worldwide were attributable to insufficient fruits and vegetables consumption (3.9 million deaths, of which 2.4 million and 1.5 million were due to low fruit and vegetable intake, respectively), including oral cancer, esophageal cancer, lung cancer, Type 2 diabetes, and various cardiovascular and cerebrovascular diseases [7]. On the other hand, studies have shown that increasing fruit and vegetable intake can reduce the risk of many chronic diseases. A prospective cohort study of 120,000 people in Shanghai, China, showed that high fruit consumption reduced the risk of coronary heart disease in Chinese women, with a 12% reduction in the risk of cardiovascular disease for every 80 g/d increase in intake [8]. Increased intake of certain fruits was associated with lower SBP, DBP, and weight gain [9,10,11]. Intake of green leafy vegetables and cruciferous vegetables was negatively associated with the risk of diabetes, cardiovascular disease, and cancer [12,13]. In addition, several systematic reviews and meta-analysis demonstrated that high fruit and/or vegetable intake reduced cardiovascular disease morbidity and mortality, whether assessing overall fruit and vegetable intake or single fruit or vegetable intake [10,12,14,15].

To improve the health of the global population and reduce the risk of certain non-communicable diseases (NCDs), the World Health Organization (WHO) recommends consuming of at least 400 g of fruits and vegetables per day [16]. Many countries have developed their own dietary recommendations based on the WHO recommendations to increase fruit and vegetable intake. China has also developed its own recommended fruit and vegetable intake based on its national conditions, with different recommendations for different age groups. According to the latest version of the Chinese Dietary Guidelines (2022), the recommended daily intake of fruits and vegetables for adults is 200–350 g and 300–500 g, respectively [17]. So far, there are increasingly studies on the dietary intake of Chinese residents, but there are not many systematic researches focusing on the fruit and vegetable intake among adult labor force population.

In this study, we presented the status of fresh fruit and fresh vegetable consumption among the Chinese adult labor force (18–64 years old), using data from the latest nationally representative cross-sectional survey, the China Nutrition and Health Surveillance, 2015–2017 (CNHS 2015–2017). We also analyzed the differences in subgroups and influencing factors under different socio-demographic characteristics, and explored the association between fruit and vegetable intake and malnutrition. By synthesizing the results, we aim to provide a basis for the development of intervention plans for fruit and vegetable intake in the Chinese labor force, and contribute to the establishment of a reasonable and correct dietary structure for this population. 

## 2. Data Sources and Methods

### 2.1. Study Design

The data were from the China Nutrition and Health Surveillance, 2015–2017 [18]. It was a cross-sectional study and led by the National Health Commission of the People’s Republic of China. In 2015, the target population of the surveillance was adults ≥18 years old. A multistage stratified systematic clustered random sampling design was carried out in 31 provincial-level administrative divisions (PLADs) in mainland China and a total of 302 survey sites (counties/districts/Xinjiang Production and Construction Corps) were randomly selected nationwide. At least 270 households were selected at each survey site, including at least 612 permanent residents aged ≥18 years old. The sample was nationally representative, as well as regionally (eastern, central, and western), urban–rural, and provincially representative. The study was launched in June 2015, and after training for investigators at all levels and completion of sampling and other preliminary work, on-site surveys were conducted in 31 provinces during the same period from August to November. Nutrition and health-related indicators were collected in the Chinese population through interviews, physical measurements, laboratory tests, and dietary surveys. The study protocol was evaluated and approved by the Ethical Committee of the Chinese Center for Disease Control and Prevention (China CDC) (No. 201519-B). All the information was collected by trained investigators and participants in this survey voluntarily participated and signed the informed consent.

Survey information from people aged 18 to 64 years old who participated in dietary survey was extracted for in-depth analysis in this study. Abnormal dietary energy intake was removed according to the interquartile range (IQR) test (the 2.5th and the 97.5th quantiles). Missing data on sociodemographic information, dietary intake, and anthropometric measurements were excluded during the process of data processing. A total of 45,459 adults were included in this study.

### 2.2. Data Collections

The CNHS 2015–2017 applied standardized questionnaires and measurements to collect detailed information for demographic and socioeconomic information, dietary behaviors, and anthropometric indexes.

Individual essential information such as age, gender, residential area, educational attainment, occupation, household income, and marital status was obtained through face-to-face interviews by basic information questionnaire.

Fruits and vegetables consumption data were assessed by face-to-face interview using a structured food frequency questionnaire (FFQ). Participants were asked to recall the frequency of their intake of all fruits and vegetables in the past 12 months (recorded as times/day, times/week, times/month, or times/year depending on the frequency of intake), while recording the average amount consumed each time (edible part). During the recall process, the investigator used food scale and size-referenced food chart (with 21 examples of the most common fruits and 23 examples of vegetables on the market for reference) to help the participants estimate their intake. Fruit means fresh fruits except fruit juice, dried fruits, preserved fruits, and canned fruits. Vegetable means fresh vegetables, excluding dried vegetables, preserved vegetables and potatoes, cassava, and other tubers. 

Physical measurements were taken according to standardized techniques and all equipment was calibrated before use. For height measurements, each monitoring site uses the same brand and type of height meter (TZG) to measure on a solid horizontal ground, and the count is accurate to 0.1 cm. Weight was measured to an accuracy of 0.1 kg, using an electronic scale (TANITA, HD-390).

### 2.3. Fruits and Vegetable Intake Assessment

The consumptions of fruits and vegetables were evaluated according to the recommended intake in the Dietary Guidelines for Chinese Residents (2022). The recommended daily intake of fruit and vegetable for people aged 18–64 years old are 200–350 g and 300–500 g, respectively [17]. The consumption of combined fruit and vegetable were evaluated according to the World Health Organization (WHO)’s recommendation: the daily minimum intake of fruits and vegetables is 400 g [16]. 

Daily energy-adjusted intakes of fruits and vegetables (grams per 1000 Kcal, g/1000 Kcal) were also calculated based on the daily total energy intake and categorized into four grades according to the quartile distribution. For fruits, Q1 < 11.32 g/1000 Kcal, 11.32 ≤ Q2 < 35.46 g/1000 Kcal, 35.46 ≤ Q3 < 78.78 g/1000 Kcal, and Q4 ≥ 78.78 g/1000 Kcal. For vegetables, Q1 < 74.17 g/1000 Kcal, 74.17 ≤ Q2 < 132.9 g/1000 Kcal, 132.9 ≤ Q3 < 217.51 g/1000 Kcal, and Q4 ≥ 217.51 g/1000 Kcal. Daily energy intake of participants was calculated based on the China Food Composition tables [19,20,21].

### 2.4. Weight Status and Malnutrition Diagnosis 

The BMI was calculated as BMI = weight/height^2^ (kg/m^2^) and weight status was classified according to the Health Industry Standards of China, Criteria of Weight for Adults (WS/T 428–2013) [22].

(1) Underweight: BMI < 18.5;
(2) Normal weight: 18.5 ≤ BMI < 24.0;
(3) Overweight: 24.0 ≤ BMI < 28.0;
(4) Obesity: BMI ≥ 28.0.

Malnutrition in this study refers to both undernutrition and overnutrition, including underweight, overweight, and obesity.

### 2.5. Covariates

The study participants were grouped according to gender, age, residential area, regional division, educational level, occupation, household income (household income per capita in 2014), and marital status.

All the participants were divided into four age groups: 18–29, 30–39, 40–49, and 50–64 years old. The residential area was classified as urban and rural based on administrative division according to the National Bureau of Statistics of China [23]. Given the large size of the Chinese territory, we considered two different forms of geographical distribution. The regional division 1 was classified as eastern, central, and western based on geographic location from east to west according to the China Health Statistical Yearbook (eastern: Beijing, Tianjin, Hebei, Liaoning, Shanghai, Jiangsu, Zhejiang, Fujian, Shandong, Guangdong, Hainan; central: Jilin, Heilongjiang, Shanxi, Anhui, Jiangxi, Henan, Hubei, Hunan; western: Inner Mongolia, Guangxi, Chongqing, Sichuan, Guizhou, Yunnan, Tibet, Shaanxi, Gansu, Qinghai, Ningxia, Xinjiang) [24]. The regional division 2 used the Qinling Mountain–Huaihe River Line that divides China into northern and southern regions, which differ markedly in terms of climate, vegetation, and diet (northern: Beijing, Tianjin, Shanxi, Liaoning, Jilin, Heilongjiang, Hebei, Inner Mongolia, Shandong, Henan, Shaanxi, Gansu, Qinghai, Ningxia, Xinjiang; southern: the remaining 16 provinces).

Educational level was categorized into four groups: primary school and below (PS and below), junior high school (JS), high school and technical school (HS and TS), and college or above (C or above). Occupation was grouped into employed and not employed (economically inactive persons such as student, homemaker, unemployed, and retired). Household income was classified into four groups by quartiles: low (<5000 RMB), middle (≥5000 and <10,000 RMB), upper-middle (≥10,000 and <20,000 RMB), and high (≥20,000 RMB). Marital status was classified as married (married/cohabitating) and not married (unmarried/widowed/divorced/separated).

### 2.6. Statistical Analysis

All data cleaning and statistical analyses were undertaken using SAS 9.4 (SAS Institute Inc., Cary, NC, USA). PROC SURVEYFREQ and PROC SURVEYLOGISTIC were used for weight adjustment to maintain the representativeness of the sample. The sample weights were calculated based on the Sixth National Population Census of China in 2010. Quantitative data such as the consumptions of fruits and vegetables (non-normally distributed) were presented as the median (M) and interquartile range (IQR). Wilcoxon rank-sum test was performed to investigate the daily consumption between subgroups. Categorical data were expressed as percentages and logistic regression analysis was performed to adjust for confounding factors. Multivariate logistic regression analysis was used to evaluate the statistical significance of differences between subgroups and to identify factors significantly associated with fruit and vegetable intake. This analysis was also used to evaluate the associations between fruit and vegetable intakes and BMI. Odds ratios (OR), 95% confidence intervals (CI), and *p* values were calculated. Two-sided *p* values < 0.05 were considered to be statistically significant.

## 3. Results

### 3.1. General Characteristics of Participants

As shown in Table 1, a total of 45,459 labor force aged 18–64 years old from urban (40.3%) and rural (59.7%) areas of China were included in the analysis, including 21,098 males (46.4%) and 24,361 females (53.6%). According to the geographic distribution from east to west, 37.5% lived in the eastern region, 29.1% lived in the central region, and 33.4% lived in the western region. For north–south distribution, 48.7% lived in the northern region and 51.3% lived in the southern region. For further analysis, we divided the samples into four age groups, four education levels, two occupation statuses, four income levels, two marital statuses, and four BMI categories. Most of the labor force were 40 years and older (75.0%). A total of 76.6% had an education level below high school, and the majority of the respondents were employed (77.7%) and married (93.4%). According to BMI, the proportions of underweight, normal weight, overweight, and obesity were 3.3%, 46.1%, 35.7%, and 14.8%, respectively. 

### 3.2. Fruit and Vegetable Intake among Chinese Labour Force Aged 18–64

The distribution of daily intake among the labor force concerning different sociodemographic characteristics is presented in Table 2. In 2015, the daily intakes of fresh fruit, fresh vegetables, and combined fruit and vegetables among the Chinese labor force were 64.3 (128.6) g, 210.0 (280.0) g, and 330.0 (299.5) g, respectively. The intake of fresh fruit was higher in women than in men, which decreased significantly with age. Conversely, the intake of fresh vegetables was higher in men than in women and increased with age. Urban residents ate more fruits and vegetables than rural residents, and consumption of both was higher with education and income. People in the eastern region had significantly higher fruit and vegetable intake than those in the western and central regions, while the central region had the lowest fruit intakes. Fruit consumption was higher in the northern region than in the southern region, but vegetable consumption was significantly higher in the southern region. Although the median fruit intakes of the married and unmarried populations were close, the Wilcoxon rank-sum test revealed a significant group difference. Additionally, we observed an increase in fruit and vegetable intake with increasing BMI when respondents were classified into four BMI categories. 

### 3.3. Insufficient Fruit and Vegetable Intake Status among Chinese Labor Force Aged 18–64

Table 3 shows the probability of insufficient fruit and vegetable intake for the Chinese labor force. Compared with the Dietary Guidelines for Chinese Residents (2022), 79.9% of the population were at risk for insufficient fruit intake, higher among men than women. Whereas 53.0% were at risk for insufficient vegetable intake, higher among women than men. Compared with the current WHO recommendation, 55.2% of the labor force had an insufficient intake of combined fruit and vegetables. The risk of insufficient intake was high across all subgroups, especially for fruit consumption. The proportion of insufficient fruit intake was higher in rural areas than in urban areas (urban 75.6%, rural 84.7%), while there was no significant urban–rural difference in the proportion of insufficient vegetable intake (urban 51.3%, rural 54.8%). For different geographic locations, the proportion of insufficient fruit and vegetable intake was lower in the eastern region than in the central and western regions. The proportion of inadequate fruit intake among the labor force population in the northern region is lower than in the southern region, while the opposite is true for vegetables. Additionally, the proportion of insufficient fruit and vegetable intake decreased with increasing levels of education and household income. Interestingly, the proportion of insufficient vegetable intake showed a different trend from fruit in the age subgroup. The proportion of insufficient fruit intake increased significantly with age, whereas the proportion of insufficient vegetable intake decreased with age. In the categories of marital status and weight status, there were no significant differences between subgroups in the proportion of inadequate intake of fruits, vegetables, and combined fruits and vegetables.

### 3.4. Factors Associated with Fruit and Vegetable Intake among Chinese Labor Force

The odds ratio of factors associated with fruit and vegetable intake was calculated using multivariate logistic regression analysis after adjustment for confounders (Table 4). The dependent variable was adequate intake according to the Dietary Guidelines for Chinese Residents (2022). 

The model of fruit intake showed that women (OR = 1.73, 95% CI: 1.54–1.94, *p* < 0.0001) were more likely to meet the recommended fruit intake than men. Compared with primary school and below, having an educational level of junior high school (OR = 1.70, 95% CI: 1.49–1.94, *p* < 0.0001), high school and technical school (OR = 2.32, 95% CI: 1.97–2.74, *p* < 0.0001), and college (OR = 2.56, 95% CI: 2.09–3.14, *p* < 0.0001) was associated with reaching the recommended intake. Moreover, the odds of intake satisfaction was increased in upper-middle income (OR = 1.26, 95% CI: 1.06–1.49, *p* = 0.0075) and high income (OR = 1.71, 95% CI: 1.43–2.05, *p* < 0.0001) individuals compared to low-income individuals. On the contrary, the 40–49 (OR = 0.85, 95% CI: 0.72–0.99, *p* = 0.0422) and 50–64 (OR = 0.77, 95% CI: 0.65–0.91, *p* = 0.0022) age groups were significantly less likely to meet the recommendation compared with the 18–29 years age group. Living in rural areas (OR = 0.83, 95% CI: 0.73–0.93, *p* = 0.0019), central region (OR = 0.70, 95% CI: 0.61–0.79, *p* < 0.0001), western region (OR = 0.80, 95% CI: 0.70–0.90, *p* = 0.0004), and southern region (OR = 0.67, 95% CI: 0.60–0.75, *p* < 0.0001) was also associated with lower odds of reaching the recommended amount.

For vegetable intake, older age increased the odds of adequate intake (for age group 30–39, OR = 1.19, 95% CI: 1.00–1.41, *p* = 0.0476; for age group 40–49, OR = 1.27, 95% CI: 1.11–1.46, *p* = 0.0005; for age group 50–64, OR = 1.38, 95% CI: 1.19–1.61, *p* < 0.0001). Living in the southern region (OR = 1.53, 95% CI: 1.35–1.75, *p* < 0.0001) was also a protective factor for meeting the recommendation. According to education and income level, high school and technical school education (OR = 1.22, 95% CI: 1.05–1.41, *p* = 0.0079) and high income (OR = 1.17, 95% CI: 1.00–1.38, *p* = 0.0489) were associated with higher odds of adequate intake. However, no such statistically significant associations were observed in other education and income subgroups. In contrast, female gender (OR = 0.90, 95% CI: 0.83–0.98, *p* = 0.0163), living in the central (OR = 0.65, 95% CI: 0.55–0.76, *p* < 0.0001) and western (OR = 0.59, 95% CI: 0.50–0.68, *p* < 0.0001) regions, and not being employed (OR = 0.80, 95% CI: 0.71–0.90, *p* = 0.0003) were risk factors for vegetable intake. 

### 3.5. The Association between Fruit and Vegetable Intake and Malnutrition among Chinese Labor Force

The association results of fruit and vegetable intake with underweight, normal weight, overweight, and obesity are shown in Table 5. The daily intake of the survey participants was adjusted with respect to the energy intake and was divided into four classes according to the interquartile distribution. Taking into account socioeconomic level and gender differences, the urban–rural male and female populations were analyzed separately. 

After adjusting for covariates, fruit intake at the Q3 (OR = 0.40, 95% CI: 0.22–0.72) and Q4 (OR = 0.43, 95% CI: 0.24–0.79) levels might significantly reduce the risk of underweight in urban female labor force compared to the Q1 level (*p* = 0.0104). 

When fruit intake was at the Q3 level, the odds of overweight in rural males tended to increase (OR = 1.27, 95% CI: 1.04–1.54), while fruit intake at the Q4 and Q2 levels may be associated with obesity among males in urban areas as well as males and females in rural areas (urban male: Q4 vs. Q1, OR = 1.41, 95% CI: 1.04–1.92; rural male: Q4 vs. Q1, OR = 1.41, 95% CI: 1.08–1.84, *p* = 0.0451; rural female: Q2 vs. Q1, OR = 1.29, 95% CI: 1.00–1.65), but reached significance only in rural male.

For vegetables, intake was not associated with underweight. Intake at Q4 level (Q4 vs. Q1, OR = 1.37, 95% CI: 1.01–1.86) for urban males and Q3 level (Q3 vs. Q1, OR = 1.30, 95% CI: 1.00–1.70) for urban females increased the odds of normal weight, but intake at Q3 level (Q3 vs. Q1, OR = 0.81, 95% CI: 0.66–0.99) reduced the odds of normal weight in rural men. Furthermore, there was a negative association between vegetable intake and overweight in both urban males (Q4 vs. Q1, OR = 0.70, 95% CI: 0.51–0.97) and urban females (Q3 vs. Q1, OR = 0.75, 95% CI: 0.56–0.99). However, none of these associations reached significance.

## 4. Discussion

In this study, we analyzed the intake and the distributions of fruits and vegetables in China’s adult labor force population aged 18–64 years old using the most recent nationally representative data. The fresh fruit and vegetable intakes (median) of the Chinese labor population in 2015 were 64.3 g/d and 210 g/d, respectively. The total fresh fruit and vegetable intake (median) was 330.0 g/d. Compared with the historical data, the fresh fruit intake had slightly decreased from 2002 (45.0 g/d) to 2012 (40.7 g/d), but had a significantly increased from 2012 to 2015 [25]. However, the fruit intake among the Chinese labor force in 2015 was still at a low level compared with the global average [26], the United States [27,28], Canada [29], England [15], Australia [30], and most other high-income countries, but not much different from most neighboring Asian countries (except Japan and Korea) and higher than in sub-Saharan Africa [26,31]. For fresh vegetables, although this intake of 210 g/d is at a high level in the world [32], the trend of continuous decline from 1982 to 2015 still needs to be a cause for concern. Especially in rural areas, the decline was 37.9% and 28.1%, compared to 322 g/d in 1982 and 256.1 g/d in 2012, respectively [33]. Moreover, in Asia, where the diet is mainly plant-based, this level is higher than in most Asian countries, but lower than in Korea, Lao PDR, Japan, and Timor-Leste [32,34]. Combined with the geographical distribution, people in the southern region eat more fresh vegetables than those in the northern region, and the intake of fruit was higher among residents of the northern region, 1.7 times higher than those in the southern region, similar to other studies in China [35]. We can also note the lower intake of fruits and/or vegetables among people living in rural, central, or western region and with lower socioeconomic status (i.e., education, income). The discrepancy of intake between different parts of China may be due to different levels of economic development and living habits. The poorer diet quality of lower socioeconomic groups has also been confirmed [36,37]. Therefore, these groups should be considered as the target population to promote the intake of fruits and vegetables.

The daily fruit and vegetable intake of each respondent was assessed according to Chinese and WHO standards to determine whether their intake was satisfactory. Although the national median vegetable intake was not low, the situation was still not promising, with more than half of the labor force (53.0%) that did not meet China’s recommended daily intake. The proportion of inadequate fruit intake was 79.9%, and people from rural areas were worse than those from urban areas (difference was of about 9 percentage points). That is, only one out of every five adult labor force in China consumed enough fruit in their daily lives. The insufficient proportion increased with age, reaching 84.7% in the 50–64 age group, which was consistent with the trend in previous reports of the WHO and other population studies in China [38,39]. In rural areas, this percentage was close to 85%, and it even reached 86–88% among people with a diploma below junior high school and with the lowest household income (<5000 RMB in 2014). The very low fruit consumption leads to a comparatively low intake of fruit and vegetable intake, with only 44.8% of the population meeting the WHO recommended intake. As one of the 10th leading level 3 global risk for disability-adjusted life-years [6], fresh fruit deficiency had become a serious problem in China. According to the Global Dietary Database, China has the highest absolute number of cardiovascular disease deaths due to inadequate fruit intake among the most populous countries [40]. There is an urgent need to promote residents to increase their daily fruit intake.

To further analyze the possible factors that may affect the intake of fruits and vegetables in Chinese adult labor force, we investigated the relationship between daily intake and sociodemographic characteristics of the population. Considering that the ultimate goal of our study was to increase the population’s daily intake of fruits and vegetables to the recommended level, a regression analysis was conducted with meeting the recommended intake as the dependent variable. Our results showed that male gender, increasing age, living in rural, central, or western region, and lower education and income levels significantly reduced the odds of consuming adequate fruit intake, while insufficient vegetable intake was significantly associated with female gender, younger age, living in central or western regions, and people without employment. 

Women were about 1.7 times more likely than men to eat 200 g or more fruit per day. This result was consistent with previous studies [29,30,33,41]. This may be because women are more health-conscious than men, and women generally prefer sweet and fruity foods. We also found an interesting phenomenon that as the age of the Chinese labor force increased, fruit intake and the odds of meeting the recommendation decreased, but the results for vegetables were the opposite. We speculate that these could be relevant to the decline of chewing and digestive ability with age, given that dental problems in older age groups appear to be a strong determinant of reduced fruit and vegetable intake [42]. Traditional Chinese cooking methods can make vegetables softer and easier to chew. This may explain why older adults prefer vegetables to fruits, which must be eaten raw. This speculation requires further investigation. Residents of urban and eastern areas were more likely to consume enough fruits and vegetables, which may be the result of uneven economic development across areas. The climate and hydrology in the southern region are more conducive to plant growth, so residents have a wider variety of vegetables to choose from than those in the northern region. In addition, higher levels of education were associated with higher odds of consuming adequate fruit intake. Higher income also increased the odds of adequate fruit and vegetable intake compared with low income (<5000 RMB). Data from studies in other countries show similar conclusions, with higher fruit and vegetable consumption in higher economic groups, regardless of a country’s economic level [43,44,45]. At the same time, people with higher levels of education have greater awareness of nutritious diets and healthy lifestyles [36,46,47]. Education level and income level are positively associated with diet. In this study, we further indicated that education levels appear to have a stronger effect on daily fruit and vegetable intake than income level. Health literacy and nutrition knowledge are fundamental and important determinants of dietary behavior.

In the present study, economically inactive persons, such as students, housewives, unemployed, and retirees, were classified in one category and employed persons in another. Compared to those with a job, those without a job were less likely to eat enough vegetables (OR = 0.80, 95% CI: 0.71–0.90, *p* = 0.0003). Although there was no significant association between occupation and insufficient fruit intake (due perhaps to the too low intake), employed individuals had significantly lower fruit intake than those who do not have jobs (57.1 g vs. 74.3 g, *p* < 0.0001). Occupation is usually closely associated with education and income level [48]. However, some associations between occupation and fruit and vegetable intake remained significant in this study after adjustment for confounding factors, including educational and income levels. This may indicate that the differences in intake between employed and non-employed groups cannot be explained by education and income levels alone, which is similar to the studies of Tanaka et al. [49]. In addition, the dietary intake of employed people is not always better than that of non-employed people. Long working hours, shift work, and heavy physical work have been confirmed to correlate with poor dietary quality and inadequate intake of micronutrients [49,50,51]. The impact of frequent canteen meals on dietary patterns also remains controversial [52,53], and the availability and accessibility of healthy foods in schools and workplaces deserves special attention [54,55]. Given the potential for differences in dietary outcomes across occupations, a more detailed classification of occupations will be conducted in future studies.

The relationship between food intake and malnutrition has been studied frequently in recent years, focusing mainly on overnutrition. Weight status is often seen as a reflection of malnutrition, with undernutrition manifested as underweight, and overnutrition manifested as overweight and obesity. Overweight and obesity is commonly thought to be associated with low intake of fruit and vegetables [56,57]. Adherence in fruit and vegetable intake was associated with lower BMI, even among overweight or obese individuals. Typically, heavier individuals tend to eat more at each meal, so direct use of daily intake when evaluating weight-related indicators may lead to bias. In this study, we used daily energy-adjusted intake rather than daily intake to evaluate the associations between fruit and vegetable intake and malnutrition, and our results suggest that increasing vegetable intake may help maintain a normal BMI among labor force living in urban areas. The inverse association between vegetables and overweight in urban areas is consistent with previous studies. However, we did not find any significant association between vegetable intake and obesity. Although most reports showed that high vegetable intake was associated with a reduced risk of overweight/obesity [58,59], some suggested that high vegetable intake was associated with a higher risk of becoming overweight or obese [60,61,62]. In view of this, we believe that the relationship between vegetables and overnutrition should be interpreted with caution, and increasing vegetable intake to reduce the risk of being overweight or obese may not be applicable to China’s labor force population, especially in rural areas. 

For fruit, our studies revealed that increasing intake may help reduce the risk of underweight in urban females, but no direct benefit was found for the prevention of overweight or obesity. Moreover, higher fruit was associated with a higher risk of overweight/obesity in urban male and both genders in rural areas. The results may be unexpected, as overweight/obesity is generally thought to be associated with low intake of fruit [63,64]. We hypothesized that the main reason why our study results differ from previously published results is the low level of fruit intake in China. Research into the molecular mechanisms of fruit’s role in health has made some progress. Many studies have confirmed that the soluble fiber, vitamin C, vitamin E, carotenoids, and phytochemicals rich in fruits are significantly associated with anti-oxidation and anti-inflammatory activities, and can also regulate intestinal flora balance [12,14,65]. However, very low intakes result in a lack of substantial variation in micronutrient and phytochemical intake. The association of fruit with malnutrition may be masked by other food with higher intakes. A U.S. cohort study of women showed that the risk of being overweight or obese was modestly reduced only when fruit intake reached 2.3 servings/day or more [61]. We concluded that if the Chinese labor force wish to reduce the risk of overweight or obesity through fruit consumption, the most critical issue is to reach the appropriate level. Another reason may be related to the type of fruit most commonly consumed by the Chinese labor force. Although fruit consumption has been associated with lower risk of many diet-related diseases, different fruits vary in terms of their protective effects. Dark green and deep yellow fruits, citrus fruits, and berries are richer in vitamins and phytochemicals than other fruits and therefore have a greater contribution to health [9,66]. According to the China Agricultural Wholesale Market Association, the number one selling fruit in China is watermelon, followed by apples and grapes, with citrus in fourth place [67]. Many fruits are very high in sugar. Although the natural sugars in fruits are healthier than the added sugars in processed foods, it is clear that fruits with high sugar content are not a good choice for weight control. Residents are influenced by actors such as price, quality, and food safety when purchasing fruit. Therefore, it is necessary to optimize the market for fruit supply and to actively guide the population’s consumption of specific types of quality fresh fruit, especially for malnourished populations.

This study had several potential limitations. First, the estimations of fresh fruit and vegetable intake were based on retrospective self-reports from the past year (FFQ). Although measures were used in the survey to help improve completeness and accuracy (e.g., using electronic gram scales and food charts), recall bias may still lead to overestimation or underestimation of intake. In order to solve this problem, it will be necessary to compare by multiday 24 h recall across seasons to make intake corrections or to conduct long-term follow-up cohort studies in our future work. Second, our study is based on cross-sectional data that allow us to describe associations but are difficult to derive causal relationships based on the present findings. The relationships between fruit/vegetable intake and health status are bidirectional. Since this study had access to only one year of national dietary intake data and measured BMI at a single time point, no definitive conclusions can be drawn in this regard. However, this study helps us to gain a preliminary understanding of the potential factors and possible correlations affecting fruit and vegetable intake in the Chinese labor force, which may help to provide a basis for further research and develop targeted improvement measures in the future. Third, food intake may be influenced by many factors. In this study, we focused on the analysis of sociodemographic factors, and there was a lack of other factors such as dietary patterns and specific occupations. Physical activity indicators were also not considered when exploring the relationship between intake and overnutrition. Given the above, more relevant research is warranted in this field and it is necessary to include more factors for a comprehensive assessment.

Despite these limitations, a strength of the study was that it included a large number of participants selected from a nationwide population. The high-quality, nationally representative data increase the validity and generalizability of our findings. China is inevitably experiencing rapid aging. With the aging of the labor force, improving the health status of the labor force is a necessary condition for healthy aging, as well as an important goal of public health. As one of the key dietary factors in the global burden of disease, a comprehensive understanding of fresh fruit and vegetable intake in the Chinese labor force, whether for research or disease control purposes, must be particularly important.

## 5. Conclusions

The intake of fresh fruit and vegetables among the Chinese labor force in 2015 was significantly low, especially for fruit. Less than a quarter of the fruit intake met the recommended intake and less than half of the population consumed the recommended amount of vegetables. Our results showed that for the Chinese Labor population, increasing age and living in rural, central, or western region might be risk factors for fruit intake, while female gender, living in central or western regions, and without employment might be risk factors for vegetable intake. We also confirmed that vegetables help to maintain a normal BMI and control overweight. As an important part of healthy diet, increasing the daily intake of fresh fruits and vegetables is an important link to improve the health status of China’s labor resources, and will also be one of the key contents of relevant departments in the future.

## Figures and Tables

**Table 1:** Descriptive characteristics of the study population.

Characteristics	*N*	Percentage (%)
Total	45,459	100.0
Gender		
Male	21,098	46.4
Female	24,361	53.6
Age group (Years)		
18–29	4695	10.3
30–39	6677	14.7
40–49	19,980	44.0
50–64	14,107	31.0
Residential area		
Urban	18,321	40.3
Rural	27,138	59.7
Regional division 1		
Eastern	17,030	37.5
Central	13,228	29.1
Western	15,201	33.4
Regional division 2		
Northern	22,148	48.7
Southern	23,311	51.3
Educational level		
PS and below	19,313	42.5
JS	15,511	34.1
HS and TS	6707	14.8
C or above	3928	8.6
Occupation		
Employed	35,344	77.7
Not employed	10,115	22.3
Household income		
Low	9102	20.0
Middle	10,993	24.2
Upper-middle	13,906	30.6
High	11,458	25.2
Marital status		
Married	42,443	93.4
Not married	3016	6.6
Weight status		
Underweight	1521	3.3
Normal	20,955	46.1
Overweight	16,238	35.7
Obesity	6745	14.8

PS: primary school, JS: junior high school, HS: high school, TS: technical school, and C: college; Not employed: student/homemaker/unemployed/retired; Married: married/cohabitating, Not married: unmarried/widowed/divorced/separated; Low income: <5000 RMB, Middle income: ≥5000 and <10,000 RMB, Upper-middle income: ≥10,000 and <20,000 RMB), and High income: ≥20,000 RMB; Underweight: BMI < 18.5, Normal weight: 18.5 ≤ BMI < 24.0, Overweight: 24.0 ≤ BMI < 28.0, and Obesity: BMI ≥ 28.0.

**Table 2:** Daily consumption of fruit and vegetables of labor force in China, 2015 (g/day).

Characteristics	Fruit		Vegetable		Fruit and Vegetable	
	M (IRQ)	*p* Value	M (IRQ)	*p* Value	M (IRQ)	*p* Value
Total	64.3 (128.6)		210.0 (280.0)		330.0 (299.5)	
Gender						
Male	51.4 (114.3)	<0.0001 **	250.0 (270.0)	<0.0001 **	328.6 (301.6)	0.0566
Female	71.4 (125.7)		200.0 (285.7)		335.7 (294.7)	
Age group (Years)						
18–29	85.7 (124.3)	<0.0001 **	200.0 (300.0)	<0.0001 **	328.6 (300.0)	0.3353
30–39	85.7 (130.0)		200.0 (300.0)		342.9 (294.1)	
40–49	57.1 (130.3)		240.0 (280.0)		326.8 (300.0)	
50–64	50.0 (114.3)		250.0 (250.0)		330.0 (305.9)	
Residential area						
Urban	91.4 (145.0)	<0.0001 **	250.0 (250.0)	<0.0001 **	370.0 (310.0)	<0.0001 **
Rural	50.0 (100.0)		200.0 (300.0)		307.9 (298.5)	
Regional division 1						
Eastern	80.0 (132.4)	<0.0001 **	300.0 (250.0)	<0.0001 **	400.0 (311.4)	<0.0001 **
Central	55.7 (115.7)		200.0 (300.0)		310.0 (275.7)	
Western	57.1 (123.1)		200.0 (300.0)		300.0 (300.0)	
Regional division 2						
Northern	85.7 (131.4)	<0.0001 **	200.0 (300.0)	<0.0001 **	314.3 (314.3)	<0.0001 **
Southern	50.0 (92.9)		300.0 (240.0)		342.9 (287.8)	
Educational level						
PS and below	42.9 (88.6)	<0.0001 **	200.0 (300.0)	<0.0001 **	300.0 (291.4)	<0.0001 **
JS	70.0 (124.3)		240.0 (250.0)		345.7 (295.7)	
HS and TS	100.0 (144.3)		250.0 (250.0)		385.7 (307.1)	
C or above	114.3 (140.0)		250.0 (230.0)		400.0 (325.0)	
Occupation						
Employed	57.1 (130.3)	<0.0001 **	214.3 (280.0)	0.8196	328.6 (300.0)	0.0008 **
Not employed	74.3 (127.1)		200.0 (271.4)		342.9 (300.0)	
Household income						
Low	40.0 (90.1)	<0.0001 **	200.0 (300.0)	<0.0001 **	300.0 (297.1)	<0.0001 **
Middle	50.0 (99.5)		200.0 (300.0)		310.0 (295.6)	
Upper-middle	64.3 (128.6)		240.0 (257.1)		342.9 (293.3)	
High	100.0 (157.1)		250.0 (250.0)		400.0 (310.0)	
Marital status						
Married	64.3 (129.0)	0.003 **	214.3 (280.0)	0.0149 *	330.0 (298.9)	0.2657
Not married	64.3 (128.6)		200.0 (300.0)		328.6 (300.0)	
Weight status						
Underweight	42.9 (92.9)	<0.0001 **	200.0 (300.0)	<0.0001 **	300.0 (284.9)	<0.0001 **
Normal	57.1 (125.7)		200.0 (280.0)		324.3 (300.0)	
Overweight	64.3 (128.6)		230.0 (277.0)		342.9 (302.9)	
Obesity	77.1 (131.4)		240.0 (260.0)		350.0 (303.6)	

Fruit: fresh fruit, Vegetables: fresh vegetables; M (IRQ): median (interquartile range); * *p* < 0.05, ** *p* < 0.01 (Wilcoxon rank-sum test).

**Table 3:** Comparison between daily fruit and vegetable consumptions of Chinese labor force consumers with the recommended reference intakes (%).

Characteristics	Fruit ^a^			Vegetable ^a^			Fruits and Vegetables ^b^		
	<200 g	≥200 g ^c^	*p* Value	<300 g	≥300 g ^c^	*p* Value	<500 g	≥500 g ^c^	*p* Value
Total	79.9	20.1		53.0	47.0		55.2	44.8	
Gender									
Male	83.4	16.6	<0.0001 **	51.1	48.9	0.0005 **	55.9	44.1	0.2208
Female	76.3	23.7		54.9	45.1		54.5	45.5	
Age group (Years)									
18–29	76.7	23.3	<0.0001 **	56.8	43.2	0.0036 **	56.3	43.7	0.4275
30–39	77.7	22.3		52.3	47.7		53.8	46.2	
40–49	81.7	18.3		51.6	48.4		54.8	45.2	
50–64	84.7	15.3		50.8	49.2		56.3	43.7	
Residential area									
Urban	75.6	24.4	<0.0001 **	51.3	48.7	0.0564	51.0	49.0	<0.0001 **
Rural	84.7	15.3		54.8	45.2		59.9	40.1	
Regional division 1									
Eastern	75.1	24.9	<0.0001 **	45.8	54.2	<0.0001 **	46.6	53.4	<0.0001 *
Central	83.1	16.9		57.3	42.7		60.4	39.6	
Western	83.5	16.5		58.8	41.2		62.2	37.8	
Regional division 2									
Northern	76.5	23.5	<0.0001	58.6	41.4	<0.0001	57.0	43.0	0.0766
Southern	82.6	17.4		48.5	51.5		53.7	46.3	
Educational level									
PS and below	88.6	11.4	<0.0001 **	54.5	45.5	0.0182 *	61.3	38.7	<0.0001 **
JS	81.1	18.9		54.2	45.8		56.5	43.5	
HS and TS	74.3	25.7		49.1	50.9		50.0	50.0	
C or above	69.0	31.0		51.9	48.1		47.9	52.1	
Occupation									
Employed	80.3	19.7	0.0425 *	51.8	48.2	<0.0001 **	54.9	45.1	0.2765
Not employed	78.2	21.8		57.9	42.1		56.4	43.6	
Household income									
Low	86.9	13.1	<0.0001 **	58.4	41.6	<0.0001 **	64.8	35.2	<0.0001 **
Middle	84.1	15.9		53.8	46.2		59.1	40.9	
Upper-middle	80.7	19.3		53.4	46.6		54.6	45.4	
High	71.7	28.3		48.5	51.5		46.9	53.1	
Marital status									
Married	80.1	19.9	0.3752	52.7	47.3	0.3023	54.9	45.1	0.4302
Not married	78.6	21.4		54.8	45.2		56.6	43.4	
Weight status									
Underweight	81.4	18.6	0.1322	53.5	46.5	0.455	57.0	43.0	0.934
Normal	80.6	19.4		52.1	47.9		55.1	44.9	
Overweight	79.8	20.2		53.3	46.7		55.0	45.0	
Obesity	77.7	22.3		54.9	45.1		55.2	44.8	

Weighted; ^a^: the Dietary Guidelines for Chinese Residents (2022), daily fruit intake (200–350 g), daily vegetable intake (300–500 g); ^b^: the World Health Organization (WHO)’s recommendation, daily minimum intake of fruit and vegetables (400 g); ^c^: meeting the requirements or excessive intake; adjusted for gender, age, residential area, educational level, occupation, income level, marital status, and weight status; * *p* < 0.05, ** *p* < 0.01 (multivariate logistic regression analysis).

**Table 4:** Factors associated with fruit and vegetable intake.

Characteristics	Ref.	Fruit ^a^		Vegetable ^a^	
		OR (95% CI)	*p* Value	OR (95% CI)	*p* Value
Gender					
Female	Male	1.73 (1.54–1.94)	<0.0001 **	0.90 (0.83–0.98)	0.0163 *
Age group (Years)					
30–39	18–29	0.93 (0.78–1.11)	0.415	1.19 (1.00–1.41)	0.0476 *
40–49	18–29	0.85 (0.72–0.99)	0.0422 *	1.27 (1.11–1.46)	0.0005 **
50–64	18–29	0.77 (0.65–0.91)	0.0022 **	1.38 (1.19–1.61)	<0.0001 **
Residential area					
Rural	Urban	0.83 (0.73–0.93)	0.0019 **	0.98 (0.85–1.13)	0.7507
Regional division 1					
Central	Eastern	0.70 (0.61–0.79)	<0.0001 **	0.65 (0.55–0.76)	<0.0001 **
Western	Eastern	0.80 (0.70–0.90)	0.0004 **	0.59 (0.50–0.68)	<0.0001 **
Regional division 2					
Southern	Northern	0.67 (0.60–0.75)	<0.0001 **	1.53 (1.35–1.75)	<0.0001 **
Educational level					
JS	PS and below	1.70 (1.49–1.94)	<0.0001 **	1.04 (0.95–1.14)	0.4201
HS and TS	PS and below	2.32 (1.97–2.74)	<0.0001 **	1.22 (1.05–1.41)	0.0079 **
C or above	PS and below	2.56 (2.09–3.14)	<0.0001 **	1.05 (0.86–1.27)	0.6387
Occupation					
Not employed	Employed	1.05 (0.91–1.20)	0.5178	0.80 (0.71–0.90)	0.0003 **
Household income					
Middle	Low	1.12 (0.93–1.34)	0.2339	1.14 (1.00–1.31)	0.0534
Upper-middle	Low	1.26 (1.06–1.49)	0.0075 **	1.06 (0.92–1.22)	0.4039
High	Low	1.71 (1.43–2.05)	<0.0001 **	1.17 (1.00–1.38)	0.0489 *
Marital status					
Not married	Married	0.83 (0.68–1.03)	0.0885	0.98 (0.81–1.18)	0.8001
Weight status					
Underweight	Normal	1.10 (0.74–1.62)	0.6516	1.08 (0.85–1.38)	0.5375
Overweight	Normal	1.20 (0.81–1.79)	0.3683	1.02 (0.77–1.34)	0.9156
Obesity	Normal	1.32 (0.87–1.99)	0.1896	0.99 (0.76–1.30)	0.9662

Weighted; ^a^: the dependent variable was adequate intake according to the Dietary Guidelines for Chinese Residents (2022), fruit intake ≥ 200 g/day, vegetable intake ≥ 300 g/day; the model was adjusted for gender, age, residential area, educational level, occupation, income level, marital status and weight status; OR (95% CI): odds ratio (95% confidence interval); OR and corresponding 95% CIs are presented in bold when the 95% CI did not cross 1; * *p* < 0.05, ** *p* < 0.01 (multivariate logistic regression analysis).

**Table 5:** Association between fruit and vegetable intake levels and weight status.

	Underweight	Normal	Overweight	Obesity
	OR (95% CI)	OR (95% CI)	OR (95% CI)	OR (95% CI)
Fruit				
Urban Male				
Q1	Ref.	Ref.	Ref.	Ref.
Q2	0.74 (0.24–2.31)	0.93 (0.70–1.24)	1.03 (0.80–1.32)	1.12 (0.82–1.52)
Q3	1.59 (0.55–4.59)	1.07 (0.82–1.41)	0.85 (0.63–1.14)	1.01 (0.73–1.40)
Q4	1.41 (0.45–4.43)	0.72 (0.51–1.03)	1.02 (0.78–1.32)	1.41 (1.04–1.92)
*p* for trend	0.3437	0.0917	0.5161	0.1016
Urban Female				
Q1	Ref.	Ref.	Ref.	Ref.
Q2	0.74 (0.38–1.44)	1.03 (0.79–1.33)	1.17 (0.88–1.56)	0.80 (0.53–1.20)
Q3	0.40 (0.22–0.72)	1.10 (0.83–1.45)	1.22 (0.90–1.65)	0.80 (0.53–1.22)
Q4	0.43 (0.24–0.79)	1.07 (0.82–1.39)	1.25 (0.96–1.63)	0.82 (0.53–1.25)
*p* for trend	0.0104 *	0.8978	0.4302	0.736
Rural Male				
Q1	Ref.	Ref.	Ref.	Ref.
Q2	1.08 (0.63–1.86)	0.90 (0.75–1.06)	1.00 (0.83–1.21)	1.24 (0.98–1.57)
Q3	0.71 (0.37–1.37)	0.83 (0.68–1.00)	1.27 (1.04–1.54)	1.05 (0.78–1.42)
Q4	0.95 (0.47–1.94)	0.84 (0.67–1.06)	1.01 (0.79–1.29)	1.41 (1.08–1.84)
*p* for trend	0.608	0.2057	0.1062	0.0451 *
Rural Female				
Q1	Ref.	Ref.	Ref.	Ref.
Q2	0.79 (0.51–1.24)	0.96 (0.79–1.17)	0.94 (0.74–1.19)	1.29 (1.00–1.65)
Q3	0.83 (0.47–1.46)	0.95 (0.75–1.19)	1.03 (0.75–1.41)	1.11 (0.86–1.44)
Q4	0.77 (0.46–1.30)	1.09 (0.87–1.36)	0.87 (0.65–1.18)	1.12 (0.86–1.45)
*p* for trend	0.7238	0.3779	0.3739	0.2823
Vegetable				
Urban Male				
Q1	Ref.	Ref.	Ref.	Ref.
Q2	1.23 (0.59–2.55)	1.13 (0.86–1.50)	0.83 (0.63–1.10)	1.03 (0.74–1.43)
Q3	0.95 (0.39–2.27)	1.25 (0.93–1.66)	0.91 (0.70–1.18)	0.82 (0.58–1.16)
Q4	1.62 (0.68–3.84)	1.37 (1.01–1.86)	0.70 (0.51–0.97)	0.92 (0.65–1.31)
*p* for trend	0.1312	0.2137	0.1297	0.5014
Urban Female				
Q1	Ref.	Ref.	Ref.	Ref.
Q2	1.01 (0.51–1.98)	1.22 (0.93–1.61)	0.87 (0.63–1.20)	0.91 (0.62–1.32)
Q3	0.93 (0.46–1.87)	1.30 (1.00–1.70)	0.75 (0.56–0.99)	1.07 (0.78–1.46)
Q4	0.89 (0.44–1.81)	1.11 (0.86–1.45)	0.93 (0.72–1.22)	1.01 (0.74–1.39)
*p* for trend	0.9785	0.1873	0.1495	0.8095
Rural Male				
Q1	Ref.	Ref.	Ref.	Ref.
Q2	0.86 (0.47–1.56)	1.02 (0.86–1.21)	1.13 (0.95–1.35)	0.81 (0.64–1.01)
Q3	0.61 (0.35–1.06)	0.93 (0.76–1.15)	1.21 (0.97–1.51)	0.96 (0.74–1.24)
Q4	0.65 (0.37–1.15)	1.12 (0.92–1.37)	1.07 (0.89–1.30)	0.79 (0.61–1.03)
*p* for trend	0.2306	0.3834	0.3017	0.125
Rural Female				
Q1	Ref.	Ref.	Ref.	Ref.
Q2	0.89 (0.55–1.44)	0.94 (0.78–1.14)	1.01 (0.79–1.28)	1.15 (0.87–1.52)
Q3	1.11 (0.65–1.88)	0.81 (0.66–0.99)	1.21 (0.94–1.56)	1.04 (0.81–1.34)
Q4	0.75 (0.46–1.23)	1.02 (0.86–1.22)	1.03 (0.84–1.26)	0.99 (0.77–1.28)
*p* for trend	0.3771	0.0855	0.3895	0.664

Weighted. The daily intake was adjusted for energy intake. Fruit intake (g/1000 Kcal): Q1 < 11.32, 11.32 ≤ Q2 < 35.46, 35.46 ≤ Q3 < 78.78, Q4 ≥ 78.78; vegetable intake (g/1000 Kcal): Q1 < 74.17, 74.17 ≤ Q2 < 132.9, 132.9 ≤ Q3 < 217.51, Q4 ≥ 217.51). The model was adjusted for gender, age, residential area, educational level, occupation, income level, and marital status; OR and corresponding 95% CIs are presented in bold when the 95% CI did not cross 1; * *p* < 0.05 (multivariate logistic regression analysis).

## Data Availability

The data used in this study are not publicly available.
